# Global Characterisation of Coagulopathy in Isolated Traumatic Brain Injury (iTBI): A CENTER-TBI Analysis

**DOI:** 10.1007/s12028-020-01151-7

**Published:** 2020-12-11

**Authors:** Julia K. Böhm, Helge Güting, Sophie Thorn, Nadine Schäfer, Victoria Rambach, Herbert Schöchl, Oliver Grottke, Rolf Rossaint, Simon Stanworth, Nicola Curry, Rolf Lefering, Marc Maegele, CENTER-TBI Participants and Investigators, Cecilia Åkerlund, Krisztina Amrein, Nada Andelic, Lasse Andreassen, Audny Anke, Anna Antoni, Gérard Audibert, Philippe Azouvi, Maria Luisa Azzolini, Ronald Bartels, Pál Barzó, Romuald Beauvais, Ronny Beer, Bo-Michael Bellander, Antonio Belli, Habib Benali, Maurizio Berardino, Luigi Beretta, Morten Blaabjerg, Peter Bragge, Alexandra Brazinova, Vibeke Brinck, Joanne Brooker, Camilla Brorsson, Andras Buki, Monika Bullinger, Manuel Cabeleira, Alessio Caccioppola, Emiliana Calappi, Maria Rosa Calvi, Peter Cameron, Guillermo Carbayo Lozano, Marco Carbonara, Simona Cavallo, Giorgio Chevallard, Arturo Chieregato, Giuseppe Citerio, Iris Ceyisakar, Hans Clusmann, Mark Coburn, Jonathan Coles, Jamie D. Cooper, Marta Correia, Amra Čović, Nicola Curry, Endre Czeiter, Marek Czosnyka, Claire Dahyot-Fizelier, Paul Dark, Helen Dawes, Véronique De Keyser, Vincent Degos, Francesco Della Corte, Hugo den Boogert, Bart Depreitere, Đula Đilvesi, Abhishek Dixit, Emma Donoghue, Jens Dreier, Guy-Loup Dulière, Ari Ercole, Patrick Esser, Erzsébet Ezer, Martin Fabricius, Valery L. Feigin, Kelly Foks, Shirin Frisvold, Alex Furmanov, Pablo Gagliardo, Damien Galanaud, Dashiell Gantner, Guoyi Gao, Pradeep George, Alexandre Ghuysen, Lelde Giga, Ben Glocker, Jagoš Golubovic, Pedro A. Gomez, Johannes Gratz, Benjamin Gravesteijn, Francesca Grossi, Russell L. Gruen, Deepak Gupta, Juanita A. Haagsma, Iain Haitsma, Raimund Helbok, Eirik Helseth, Lindsay Horton, Jilske Huijben, Peter J. Hutchinson, Bram Jacobs, Stefan Jankowski, Mike Jarrett, Ji-yao Jiang, Faye Johnson, Kelly Jones, Mladen Karan, Angelos G. Kolias, Erwin Kompanje, Daniel Kondziella, Evgenios Koraropoulos, Lars-Owe Koskinen, Noémi Kovács, Ana Kowark, Alfonso Lagares, Linda Lanyon, Steven Laureys, Fiona Lecky, Didier Ledoux, Rolf Lefering, Valerie Legrand, Aurelie Lejeune, Leon Levi, Roger Lightfoot, Hester Lingsma, Andrew I. R. Maas, Ana M. Castaño-León, Marc Maegele, Marek Majdan, Alex Manara, Geoffrey Manley, Costanza Martino, Hugues Maréchal, Julia Mattern, Catherine McMahon, Béla Melegh, David Menon, Tomas Menovsky, Ana Mikolic, Benoit Misset, Visakh Muraleedharan, Lynnette Murray, Ancuta Negru, David Nelson, Virginia Newcombe, Daan Nieboer, József Nyirádi, Otesile Olubukola, Matej Oresic, Fabrizio Ortolano, Aarno Palotie, Paul M. Parizel, Jean-François Payen, Natascha Perera, Vincent Perlbarg, Paolo Persona, Wilco Peul, Anna Piippo-Karjalainen, Matti Pirinen, Horia Ples, Suzanne Polinder, Inigo Pomposo, Jussi P. Posti, Louis Puybasset, Andreea Radoi, Arminas Ragauskas, Rahul Raj, Malinka Rambadagalla, Jonathan Rhodes, Sylvia Richardson, Sophie Richter, Samuli Ripatti, Saulius Rocka, Cecilie Roe, Olav Roise, Jonathan Rosand, Jeffrey V. Rosenfeld, Christina Rosenlund, Guy Rosenthal, Rolf Rossaint, Sandra Rossi, Daniel Rueckert, Martin Rusnák, Juan Sahuquillo, Oliver Sakowitz, Renan Sanchez-Porras, Janos Sandor, Nadine Schäfer, Silke Schmidt, Herbert Schoechl, Guus Schoonman, Rico Frederik Schou, Elisabeth Schwendenwein, Charlie Sewalt, Toril Skandsen, Peter Smielewski, Abayomi Sorinola, Emmanuel Stamatakis, Simon Stanworth, Robert Stevens, William Stewart, Ewout W. Steyerberg, Nino Stocchetti, Nina Sundström, Anneliese Synnot, Riikka Takala, Viktória Tamás, Tomas Tamosuitis, Mark Steven Taylor, Braden Te Ao, Olli Tenovuo, Alice Theadom, Matt Thomas, Dick Tibboel, Marjolein Timmers, Christos Tolias, Tony Trapani, Cristina Maria Tudora, Andreas Unterberg, Peter Vajkoczy, Shirley Vallance, Egils Valeinis, Zoltán Vámos, Mathieu van der Jagt, Gregory Van der Steen, Joukje van der Naalt, Jeroen T. J. M. van Dijck, Thomas A. van Essen, Wim Van Hecke, Caroline van Heugten, Dominique Van Praag, Thijs Vande Vyvere, Roel P. J. van Wijk, Alessia Vargiolu, Emmanuel Vega, Kimberley Velt, Jan Verheyden, Paul M. Vespa, Anne Vik, Rimantas Vilcinis, Victor Volovici, Nicole von Steinbüchel, Daphne Voormolen, Petar Vulekovic, Kevin K. W. Wang, Eveline Wiegers, Guy Williams, Lindsay Wilson, Stefan Winzeck, Stefan Wolf, Zhihui Yang, Peter Ylén, Alexander Younsi, Frederick A. Zeiler, Veronika Zelinkova, Agate Ziverte, Tommaso Zoerle

**Affiliations:** 1 Department of Medicine, Faculty of Health, Institute for Research in Operative Medicine, Witten/Herdecke University, Ostmerheimer Str. 200, Building 38, 51109 Cologne, Germany; 2 Emergency and Trauma Centre, Alfred Health, 55 Commercial Road, Melbourne, VIC 3004 Australia; 3 Department of Traumatology, Orthopaedic Surgery and Sports Traumatology, Cologne-Merheim Medical Centre (CMMC), Witten/Herdecke University, Campus Cologne-Merheim, Ostmerheimer Str. 200, 51109 Cologne, Germany; 4 Department of Anaesthesiology and Intensive Care, AUVA Trauma Hospital, Academic Teaching Hospital of the Paracelsus Medical University, Doktor-Franz-Rehrl-Platz 5, 5010 Salzburg, Austria; 5 Ludwig Boltzmann Institute for Experimental and Clinical Traumatology, AUVA Research Centre, Donaueschingenstr. 13, 1200 Vienna, Austria; 6 Department of Anaesthesiology, RWTH Aachen University Hospital, Pauwelsstraße 30, 52074 Aachen, Germany; 7 NHS Blood and Transplant, Oxford University Hospital NHS Foundation Trust, Headley Way, OX3 9DU Oxford, UK

**Keywords:** CENTER-TBI, Traumatic brain injury, Coagulopathy, Risk factors

## Abstract

**Background:**

Trauma-induced coagulopathy in patients with traumatic brain injury (TBI) is associated with high rates of complications, unfavourable outcomes and mortality. The mechanism of the development of TBI-associated coagulopathy is poorly understood.

**Methods:**

This analysis, embedded in the prospective, multi-centred, observational Collaborative European NeuroTrauma Effectiveness Research in Traumatic Brain Injury (CENTER-TBI) study, aimed to characterise the coagulopathy of TBI. Emphasis was placed on the acute phase following TBI, primary on subgroups of patients with abnormal coagulation profile within 4 h of admission, and the impact of pre-injury anticoagulant and/or antiplatelet therapy. In order to minimise confounding factors, patients with isolated TBI (iTBI) (*n *= 598) were selected for this analysis.

**Results:**

Haemostatic disorders were observed in approximately 20% of iTBI patients. In a subgroup analysis, patients with pre-injury anticoagulant and/or antiplatelet therapy had a twice exacerbated coagulation profile as likely as those without premedication. This was in turn associated with increased rates of mortality and unfavourable outcome post-injury. A multivariate analysis of iTBI patients without pre-injury anticoagulant therapy identified several independent risk factors for coagulopathy which were present at hospital admission. Glasgow Coma Scale (GCS) less than or equal to 8, base excess (BE) less than or equal to − 6, hypothermia and hypotension increased risk significantly.

**Conclusion:**

Consideration of these factors enables early prediction and risk stratification of acute coagulopathy after TBI, thus guiding clinical management.

## Introduction

Traumatic brain injury (TBI) remains a leading cause of death and disability worldwide [1]. The initial insult often results in disruptions of the cerebral vasculature and pathological alterations of the blood–brain barrier (BBB) which may evolve into haemorrhagic lesions. In addition, TBI-associated factors may disturb the body’s haemocoagulative capacity and alter the delicate balance between bleeding and thrombus formation leading to a substantial exacerbation of the initial injury sustained [2–5]. Recent evidence suggests that the acute phase after TBI is rather characterised by dysfunction of the coagulation cascade and hyperfibrinolysis, both of which likely contribute to haemorrhagic progression. This may then be followed by platelet dysfunction and decreased platelet count while the clinical implication of these alterations remains unclear. At later stages, a poorly defined prothrombotic state emerges, partly due to fibrinolysis shutdown and hyperactive platelets [6–8]. Haemostatic alterations, in particular those during the acute phase after TBI, have been associated with higher mortality and more unfavourable outcome than in non-coagulopathic TBI patients [2, 4, 9–11].

The present study aimed to further characterise the alterations to the haemostatic system occurring in the context of isolated TBI (iTBI) based upon data collected into the central database (INCF Neurobot tool version 2.0 (INCF, Stockholm, Sweden) of the prospective, multi-centred, observational Collaborative European NeuroTrauma Effectiveness Research in Traumatic Brain Injury (CENTER-TBI) cohort study. Particular interest was given to the impact of pre-injury anticoagulant and/or antiplatelet therapy. Risk stratification was performed to identify independent predictors indicating coagulopathy after iTBI.

## Methods

### Study Population

The present study was an embedded study to the longitudinal, observational CENTER-TBI study, which recruited patients from 60 selected centres across Europe and Israel between December 2014 and December 2017 [12]. A total of 4509 patients with a clinical diagnosis of TBI were included in the CENTER-TBI core database. Inclusion criteria were a clinical diagnosis of TBI, indication for CT scanning, presentation to study centre within 24 h of injury, and informed consent obtained according to local and national requirements [12]. Participants were excluded if they had any severe pre-existing neurological disorder that could have confounded outcome assessments. As part of the CENTER-TBI core study, the present analysis was performed in accordance with all relevant local and European laws. Informed consent, including the approval to use data for research purposes, was obtained from each subject according to local ethics and regulations.

Patients with extracranial injuries, defined as AIS_Extracranial_ > 0, and those missing critical data points were excluded a priori. We included patients for whom data reporting conventional coagulation parameters within 4 h following iTBI were available. This population included subgroups that developed laboratory abnormalities and those with pre-injury anticoagulant and/or antiplatelet therapy.

### Data Collection

The cohort included patients with iTBI who were characterised with respect to the presence of haemostatic abnormalities based upon conventional coagulation parameters within 4 h of injury. The prospectively recorded parameters in scope of the CENTER-TBI core study that were considered for analysis comprised demographics, injury characteristics, medical history, medical presentation in the emergency department (ED), admission laboratory values and pre-injury anticoagulant and/or antiplatelet therapy. Follow-up data on functional outcome, including mortality and Glasgow Outcome Score-Extended (GOS-E), were obtained 6-month post-injury. A GOS-E between 1 and 4 (dead, vegetative state, low severe and upper severe disability) was considered unfavourable.

The primary outcome as the presence or absence of abnormal coagulation profile was defined by conventional coagulation parameters obtained within 4 h of the injury. The following parameters were considered for diagnosing an abnormal coagulation profile: International Normalised Ratio (INR) > 1.2 or activated partial thromboplastin time (aPTT) > 35 s or fibrinogen < 150 mg/dL or platelet count < 100 × 10^3^/nL. All relevant data for further analysis were extracted from the INCF Neurobot tool version 2.0 (INCF, Stockholm, Sweden).

### Statistical Analysis

For the descriptive analysis of iTBI patients with and without pre-injury anticoagulant and/or antiplatelet therapy, metric data are presented as median and interquartile range (IQR). Categorical data are presented in percentage. Differences were tested using the Mann–Whitney U test and Chi-squared (Chi^2^) test, respectively. Nonparametric Kruskal–Wallis test was performed to compare the standard coagulation test in relation to injury severity (AIS_Brain_) in iTBI. A *p* value < 0.05 was considered statistically significant.

In a univariate analysis, potential predictors for an abnormal coagulation profile were identified via Chi^2^ test. A logistic regression analysis (multivariate analysis) with coagulopathy as dependent variable was performed to evaluate independent risk factors associated with acute coagulopathy in iTBI. Analysis of potential predictors and independent risk factors of iTBI patients with pre-injury anticoagulant and/or antiplatelet therapy was not feasible due to the low number of cases. Some predictors were discriminated as independent risk factors (e.g. age ≥ 75, sex, neuroworsening) as no differences were detected. The predictor “arrival haemoglobin” was excluded due to low prevalence. The results are presented as odds ratio (OR) with 95% confidence interval (Cl_95_) and regression coefficient. Statistical analyses were performed using SPSS statistics version 25 for Windows (IBM Corp., Armonk, NY, USA) and GraphPadPrism version 7.00 (GraphPad Software, La Jolla California, USA).

## Results

### Cohort Characteristics

**Fig. 1:**
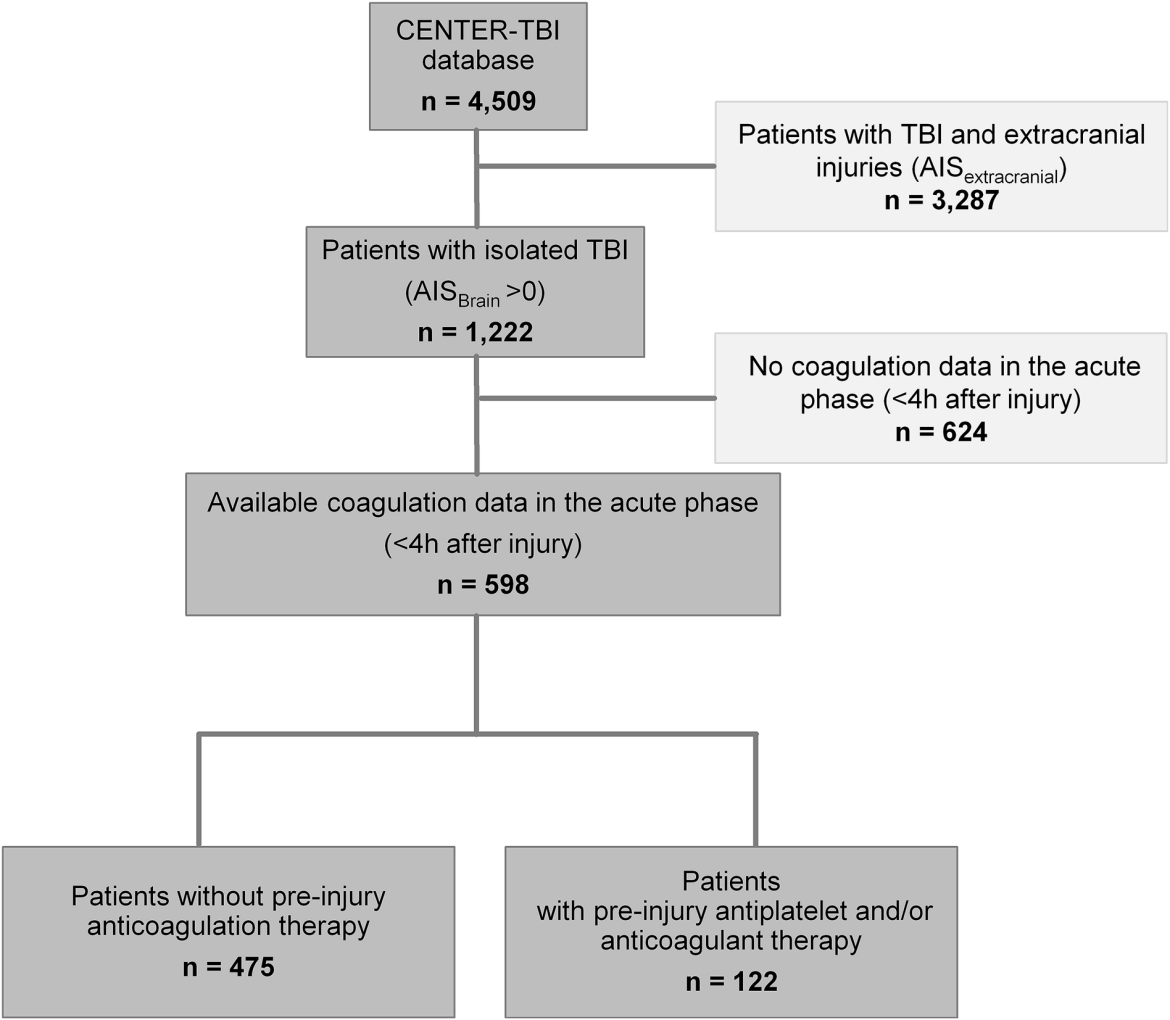
Schematic overview of CENTER-TBI cohort with inclusion and exclusion process for the present subgroup

From the 4509 patients included into the CENTER-TBI core study database, 3287 had to be excluded for co-existing extracranial injuries and 624 for missing data (Fig. 1). Thus, 598 patients with iTBI were included in the present analysis. Approximately one-fifth of the cohort was assigned to the group of elderly patients (≥ 75 years, Table 1). Almost all patients (98.7%, data not shown) had sustained a blunt trauma mechanism resulting from various injury patterns, with ground-level falls being the most common cause of injury (28.3%, data not shown). The majority of the injuries sustained were severe (AIS_Brain_ ≥ 3, 85%, Table 1) and closed head injuries (93.5%, Table 1). Computed tomography (CT) scans performed immediately after emergency department (ED) admission revealed the following most frequent intracranial pathologies: (1) subarachnoid haemorrhage (52%), (2) subdural haematoma (46.4%), (3) midline shift (24.9%), (4) extradural haematoma (16.5%), (5) basal cistern compression (13.5%), (6) depressed skull fracture (13.2%) and (7) diffuse axonal injury (9.2%) (data not shown).

**Table 1:** Characteristics of patients with isolated traumatic brain injury < 4 h following injury (*n *= 598)

	iTBI patients *n *= 598
Demographics	
Age, years; median [IQR]	52 [30–69]
Age ≥ 75; *n* [%]	106 [17.7]
Male gender; *n* [%]	415 [69.4]
Injury characteristics	
Closed TBI; *n* [%]	559 [93.5]
AIS_Brain_ 2; *n* [%]	71 [11.9]
AIS_Brain_ 3; *n* [%]	205 [34.3]
AIS_Brain_ 4; *n* [%]	158 [26.4]
AIS_Brain_ 5; *n* [%]	147 [24.6]
AIS_Brain_ 6; *n* [%]	17 [2.8]
Medical presentation at admission (ED)	
GCS; median [IQR]	14 [10–15]
SBP; mmHg; median [IQR]	138 [121–156]
Heart rate; bpm; median [IQR]	80.0 [70.5–95.0]
Temperature; °C; median [IQR]	36.2 [35.8–36.7]
Received emergency surgical intervention; *n* [%]	119 [19.9]
Coagulation status, tests and medications	
Coagulopathy; *n* [%]	117 [19.6]
Haemoglobin; g/dl; median [IQR]	13.7 [12.6–14.7]
INR; median [IQR]	1.04 [1.00–1.15]
aPTT; seconds; median [IQR]	28.2 [25.1–32.4]
Platelets;/nl; median [IQR]	224 [183–267.5]
Fibrinogen; mg/dl; median [IQR]	274.5 [230–320]
Pre-injury antiplatelet/anticoagulant medication; *n* [%]	122 [20.4]
Outcomes	
Death [overall]; *n* [%]	98 [16.4]
GOS-E [6 months—derived]; median [IQR]	7 [3–8]

*AIS* Abbreviated Injury Scale, *aPTT* activated partial thromboplastin time, *ED* Emergency department, *GCS* Glasgow Coma Scale, *GOS*-*E* Glasgow Outcome Score-Extended, *INR* International Normalized Ratio, *SBP* Systolic blood pressure

Haemostatic alterations based upon conventional coagulation parameters within 4 h after injury were present in 19.6% of included iTBI patients (*n *= 117/598, Table 1). In addition, for one in five patients pre-injury anticoagulant and/or antiplatelet therapy was documented (Table 1). Ninety-eight iTBI patients (16.4%, Table 1) died while the median outcome in surviving patients at 6 months after iTBI was favourable (Table 1).

### Subgroup Analysis of Patients with Pre-injury Antiplatelet and/or Anticoagulant Therapy

**Fig. 2:**
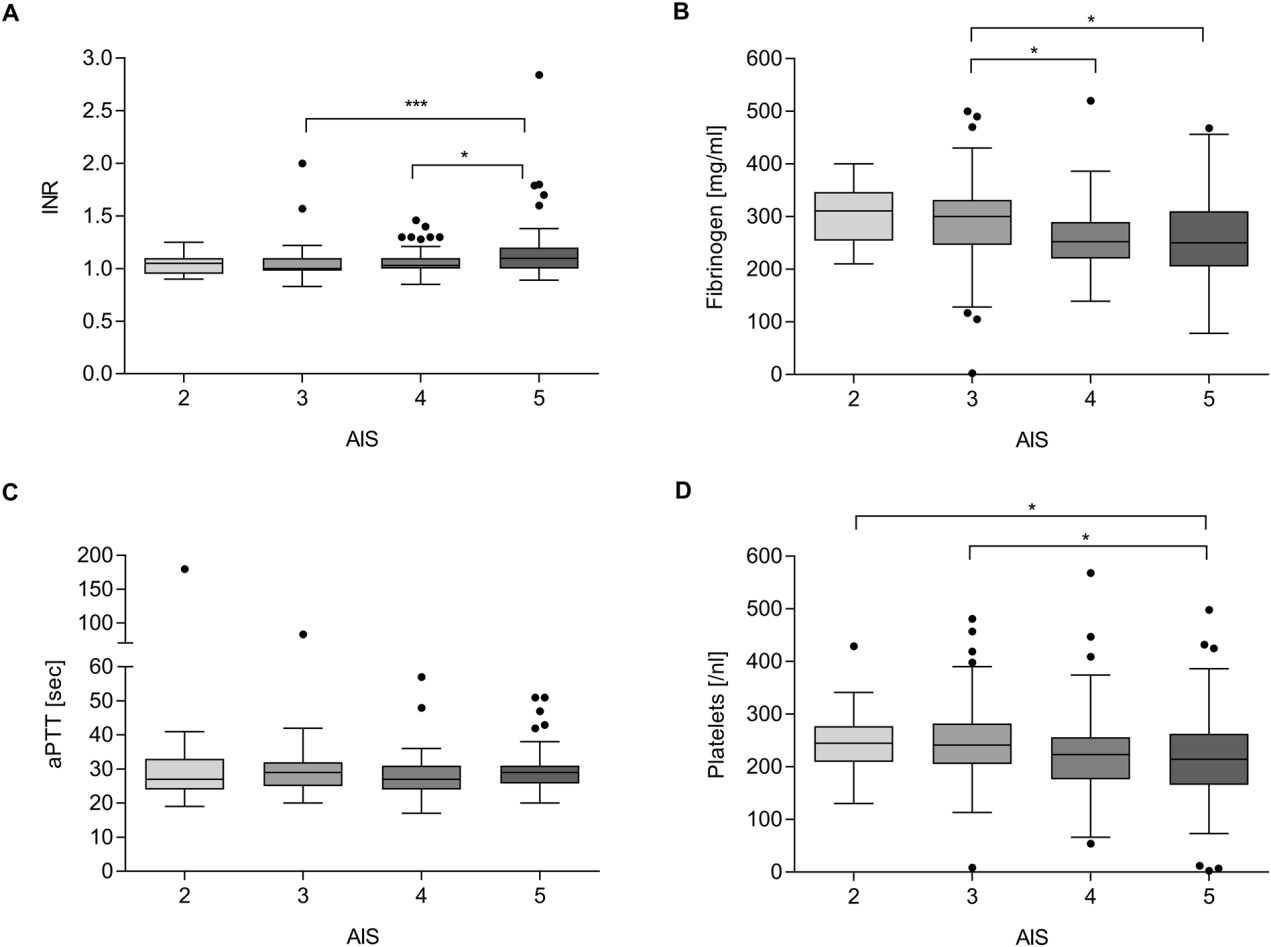
Conventional coagulation parameters INR (**a**), fibrinogen level (**b**) aPTT (**c**) and platelet count (**d**) in relation to injury severity (AIS_Brain)_ of iTBI patients (*n *= 475). One patient with AIS_Brain_ = 6 was excluded from the analysis. Statistically significant differences are marked with asterisks (*p < 0.05, **p < 0.001, ***p < 0.0001)

**Fig. 3:**
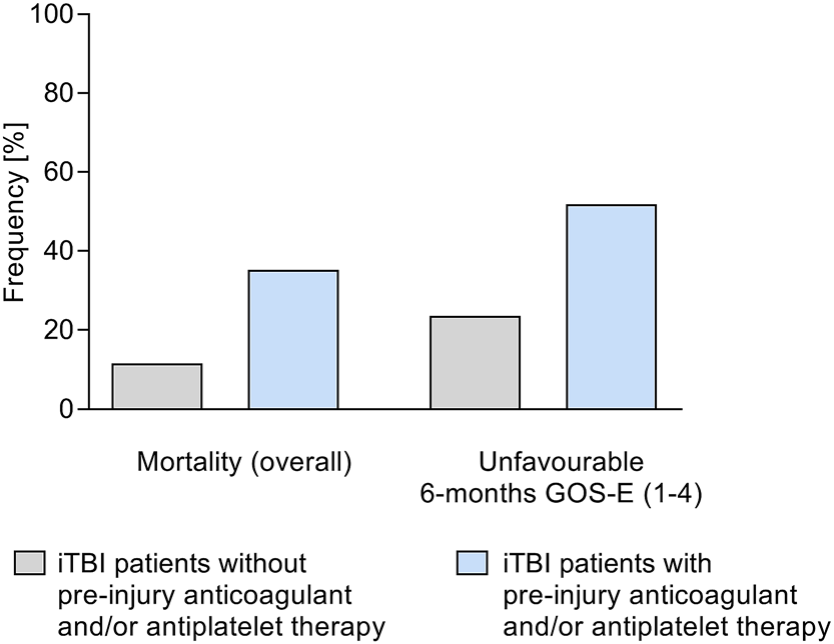
Incidence of mortality and unfavourable Glasgow Outcome Score-Extended (GOS-E) (1–4) 6-month post-injury in iTBI patients with no pre-injury anticoagulation therapy (*n *= 475) versus patients with pre-injury anticoagulation therapy (*n *= 122)

Patients with pre-injury anticoagulant and/or antiplatelet therapy were significantly older than those without. The proportion of patients ≥ 75 years of age comprised more than half in the group of patients on pre-injury anticoagulant and/or antiplatelet therapy (Table 2). A greater proportion of patients with pre-injury anticoagulant and/or antiplatelet medication had an untreatable TBI defined as AIS_Brain_ = 6. Coagulopathy by conventional coagulation parameters was diagnosed twice as frequently in patients on pre-injury anticoagulant and/or antiplatelet therapy (Table 2). Conventional coagulation parameters such as INR and aPTT were significantly deteriorated and platelet counts trended to decrease among patients with pre-injury anticoagulation and/or antiplatelet therapy (Table 2). In those patients without pre-injury anticoagulant therapy, conventional coagulation parameters significantly deteriorated with increasing severity of brain injury; a higher AIS_Brain_ correlated with higher INR, lower fibrinogen levels and lower platelet counts (Fig. 2). Patients with iTBI and on pre-injury anticoagulant and/or antiplatelet therapy had threefold higher mortality and higher frequency of unfavourable 6-month outcomes (GOS-E 1–4) compared to those without pre-injury anticoagulant and/or antiplatelet therapy (51.9% vs. 23.5%) (Table 2, Fig. 3). Notably, a higher percentage of patients with pre-injury intake of vitamin K antagonists had an abnormal coagulation profile after iTBI than patients on other pre-injury anticoagulant or antiplatelet therapy (Table 3).

**Table 2:** Characteristics of iTBI patients with and without pre-injury anticoagulation therapy (*n *= 598)

	iTBI patients without pre-injury antiplatelet and/or anticoagulant therapy *n *= 475	iTBI patients with pre-injury antiplatelet and/or anticoagulant therapy *n *= 122	*p* value
Demographics			
Age; years; median [IQR],	44 [25–61]	75 [68–81]	< 0.001
Age ≥ 75; *n* [%]	42 [8.8]	64 [52.5]	< 0.001
Male gender; *n* [%]	333 [70.1]	82 [67.2]	0.536
Injury characteristics			
Closed TBI; *n* [%]	443 [93.2]	115 [94.2]	0.690
AIS_Brain_ 2; *n* [%]	55 [11.5]	16 [13.1]	0.640
AIS_Brain_ 3; *n* [%]	164 [34.5]	41 [33.6]	0.849
AIS_Brain_ 4; *n* [%]	130 [27.4]	28 [23.0]	0.324
AIS_Brain_ 5; *n* [%]	118 [24.8]	28 [23.0]	0.665
AIS_Brain_ 6; *n* [%]	8 [1.7]	9 [7.4]	0.001
Medical presentation at admission (ED)			
GCS; median [IQR]	14 [11–15]	14 [9–15]	0.747
SBP; mmHg; median [IQR]	135 [120–150]	150 [132.5–169.2]	< 0.001
Heart rate; bpm; median [IQR]	80 [72–95]	80 [67.8–92.3]	0.368
Temperature; °C; median [IQR]	36.2 [35.8–36.7]	36.3 [35.8–36.7]	0.581
Received emergency surgical intervention; *n* [%]	97 [20.4]	21 [17.2]	0.427
Coagulopathy, standard laboratory			
Coagulopathy; *n* [%]	75 [15.8]	42 [34.4]	< 0.001
Haemoglobin; g/dl; median [IQR]	13.9 [12.7–14.8]	13.7 [12.7–14.9]	0.993
INR; median [IQR]	1.03 [1.0–1.1]	1.1 [1.0–2.48]	< 0.001
aPTT; seconds; median [IQR]	28.0 [25.0–32.0]	29.2 [26.0–35.0]	0.007
Platelets;/nl; median [IQR]	226 [183–272]	214 [185–254]	0.052
Fibrinogen; mg/dl; median [IQR]	270 [230–316.5]	304 [251.7–380]	0.018
Outcomes			
Death [overall]; *n* [%]	55 [11.6]	43 [35.2]	< 0.001
GOS-E [6 months—derived]; median [IQR]	7 [5–8]	4 [1–8]	< 0.001

Data on the presence of pre-injury anticoagulation therapy were missing in one case

*AIS* Abbreviated Injury Scale, *aPTT* activated partial thromboplastin time, *ED* Emergency department, *GCS* Glasgow Coma Scale, *GOS*-*E* Glasgow Outcome Score-Extended, *INR* International Normalized Ratio, *SBP* Systolic blood pressure

**Table 3:** Overview of pre-injury anticoagulant and/or antiplatelet therapy in iTBI patients (*n *= 122)

	iTBI patients with pre-injury antiplatelet and/or anticoagulant therapy *n *= 122	Coagulopathy *n*; [%]
Anticoagulants		
Vitamin K antagonists	37	31 [84]
Heparin	2	1 [50]
Direct oral anticoagulants (DOACs)	12	2 [17]
Other anticoagulants	4	1 [25]
Platelet inhibitors		
ASS	43	4 [9]
Other platelet inhibitors*	23	3 [13]

Anticoagulants were defined as Vitamin K antagonist (Coumarin derivates Coumadin or Warfarin), direct oral anticoagulants (Factor Xa inhibitor (e.g. Xarelto, Rivaroxaban), direct thrombin inhibitors (e.g. Dabigatran) and antithrombin protein inhibitor (e.g. ATryn). Platelet inhibitors mainly included acetylsalicylic acid (ASS). Patient specified with “Other” received platelet aggregation inhibitor such as Clopidogrel or Parasugrel. Data about specific pre-injury antiplatelet and/or anticoagulant therapy were missing for one case

*Patients with dual platelet inhibitor therapy

### Risk Factors for Coagulopathy of Patients Without Pre-injury Antiplatelet and/or Anticoagulant Therapy

Univariate analysis identified higher magnitude of brain injury (AIS_Brain_) (*p *= 0.001) and lower GCS on admission as potential independent risk factors (*p *< 0.001) for an acute coagulopathy (Table 4). Patients with coagulopathy were three times as likely to have unreactive pupils than non-coagulopathic patients with (Table 4). Coagulopathic patients were three times more likely to be hypoxic (patients with a PaO_2_ < 8 kPa (60 mmHg) and/or a SaO_2_ < 90%), eight times more likely to be hypotensive and more than five times more likely to be hypothermic (Table 4). Altered base excess (BE) (≤ − 6) occurred 5.7 times more frequently in coagulopathic patients (Table 4). Severe intracranial lesions causing basal cistern compression and severe contusions were associated with coagulopathy, with 2.5- and 2.4-fold increased incidence, respectively, among coagulopathic patients (Table 4). Mortality among coagulopathic patients with iTBI was almost three times higher than those with normal coagulation profile (25.3% vs. 9.0%; *p *< 0.0001) (data not shown). Multivariate regression analysis identified significant independent risk factors associated with coagulopathy in iTBI patients including odds ratios (OR): the GCS ≤ 8 at hospital admission had an OR of 2.4 and unbalanced BE (≤ − 6) had an OR of 3.1 (Table 5). Systemic secondary insults such as hypotension (< 90 mmHg SBP), which had an OR of 3.5 and hypothermia (temperature < 35 °C), with an OR of 2.9, were also identified (Table 5).

**Table 4:** Univariate analysis of potential risk factors associated with acute coagulopathy following iTBI of patients without pre-injury antiplatelet and/or anticoagulant therapy (*n *= 475)

	No coagulopathy *n *= 400	Coagulopathy *n *= 75	*p* value
Demographics			
Age ≥ 75; *n* [%]	38 [9.5]	4 [5.3]	0.243
Male gender; *n* [%]	283 [70.8]	50 [66.7]	0.478
Injury characteristics			
AIS_Brain_ severity			0.001
AIS 2; *n* [%]	50 [12.5]	5 [6.7]	
AIS 3; *n* [%]	143 [35.8]	21 [28.0]	
AIS 4; *n* [%]	115 [28.7]	15 [20.0]	
AIS ≥ 5; *n* [%]	92 [23.0]	34 [45.3]	
Medical presentation at admission (ED)			
GCS on admission			< 0.001
GCS ≥ 8; *n* [%]	249 [62.3]	25 [33.3]	
GCS ≤ 8; *n* [%]	49 [12.3]	22 [29.3]	
GCS unknown; *n* [%]	102 [25.5]	28 [37.3]	
Pupils [uni- or bilateral unreactive]; *n* [%]	29 [7.2]	17 [22.7]	< 0.001
Hypoxia; *n* [%]	13 [3.3]	7 [9.3]	0.016
Hypotension; *n* [%]	6 [1.5]	9 [12.0]	< 0.001
Hypothermia; *n* [%]	10 [2.5]	10 [13.3]	< 0.001
Neuroworsening; *n* [%]	48 [12.0]	8 [10.7]	0.742
Laboratory tests			
Arrival haemoglobin < 11; *n* [%]	16 [4.0]	3 [4.0]	0.742
Arrival Base Excess ≤ − 6; *n* [%]	16 [4.0]	17 [22.7]	< 0.001
Injuries identified on initial CT scan			
Diffuse axonal injury; *n* [%]	39 [10.2]	10 [14.1]	0.338
Extradural haematoma; *n* [%]	77 [19.5]	11 [14.7]	0.321
Subdural haematoma; *n* [%]	160 [40.4]	37 [49.3]	0.151
Subarachnoid haemorrhage; *n* [%]	208 [52.4]	43 [57.3]	0.432
Midline shift; *n* [%]	77 [19.6]	22 [29.3]	0.058
Basal cistern compression; *n* [%]	40 [10.2]	19 [25.3]	< 0.001
Depressed skull fracture; *n* [%]	52 [13.1]	15 [20.0]	0.116
Severe contusion; *n* [%]	22 [5.6]	10 [13.3]	0.016

Systemic secondary insult parameters pre-hospital/at hospital admission were defined as following: hypotension with systolic blood pressure (SBP) < 90 mmHg, hypothermia with temperature < 35 °C and hypoxia with a PaO_2_ < 8 kPa (60 mmHg) and/or a SaO_2_ < 90%. Neuroworsening was defined as follows: (1) a decrease in GCS motor score of 2 or more points; (2) a new loss of pupillary reactivity or development of pupillary asymmetry ≥ 2 mm; (3) deterioration in neurological or CT status sufficient to warrant immediate medical or surgical intervention

*AIS* Abbreviated Injury Scale, *CT* computed tomography, *ED* Emergency department, *GCS* Glasgow Coma Scale

**Table 5:** Independent risk factors associated with acute coagulopathy in iTBI of patients without pre-injury antiplatelet and/or anticoagulant therapy (*n *= 475)

	Regression coefficient	Odds ratio (CI_95_)	*p* value
Injury characteristics			
AIS_Brain_ severity			
AIS 3; *n* [%]	0.21	1.02 [0.45-2.31]	0.961
AIS 4; *n* [%]	− 0.52	0.59 [0.23–1.49]	0.267
AIS ≥ 5; *n* [%]	− 0.18	0.83 [0.30–2.29]	0.721
Medical presentation at admission (ED)			
GCS on admission			
GCS ≤ 8; *n* [%]	0.86	2.37 [1.20–4.69]	0.013
GCS unknown; *n* [%]	0.45	1.57 [0.87–2.85]	0.133
Pupils [uni- or bilateral unreactive]; *n* [%]	0.47	1.59 [0.78–3.24]	0.197
Hypoxia; *n* [%]	0.74	2.09 [0.79–5.57]	0.138
Hypotension; *n* [%]	1.25	3.51 [1.25–9.83]	0.017
Hypothermia; *n* [%]	1.06	2.89 [1.11–7.58]	0.030
Laboratory test			
Arrival base excess ≤ − 6; *n* [%]	1.13	3.11 [1.33–7.26]	0.009
No arrival base excess ≤ − 6; *n* [%]	− 0.92	0.91 [0.54–1.53]	0.729
Injuries identified on initial CT scan			
Midline shift; *n* [%]	0.50	1.65 [0.94–2.90]	0.830
Basal cistern compression; *n* [%]	− 0.009	0.99 [0.49–2.01]	0.980
Depressed skull fracture; *n* [%]	− 0.004	0.99 [0.51–1.93]	0.991
Severe contusion; *n* [%]	0.24	1.27 [0.56–2.89]	0.558

Systemic secondary insult parameters pre-hospital/at hospital admission were defined as following: hypotension with systolic blood pressure (SBP) < 90 mmHg, hypothermia with temperature < 35 °C and hypoxia with a PaO_2_ < 8 kPa (60 mmHg) and/or a SaO_2_ < 90%. In nine cases, data were missing for multivariate analysis

*AIS* Abbreviated Injury Scale, *CT* computed tomography, *ED* Emergency department, *GCS* Glasgow Coma Scale

In contrast to the univariate analysis (*p *= 0.016), hypoxia could not be identified as a risk factor in the multivariate analysis (*p *= 0.138) (Table 4, Table 5). However, hypoxia was only documented in 20 iTBI patients.

## Discussion

The characterisation of haemostatic abnormalities which occur in the context of isolated TBI informs our knowledge and may promote a more effective clinical risk assessment and management during the early course after trauma. The cohort analysed in the present study had a median age of 52 years, with almost one out of five patients being 75 years of age or older. For over 20% of the cohort pre-injury anticoagulant and/or antiplatelet agents, intake was documented. The mortality of the entire iTBI cohort was 16.4% and almost every fifth patient required an emergency surgical intervention. Overall, the presence of coagulopathy in the acute phase of iTBI based upon conventional coagulation parameters was observed in about 20% of all patients with iTBI. In previous reports, the prevalence of coagulopathy in TBI patients with and without extracranial injuries patients upon hospital admission was variable ranging from 7 to 63% [5]. The reported prevalence in all cases was highly dependent on how both TBI and coagulopathy were defined, the sensitivity of the coagulation assays used, the time point after injury at which the coagulation system was assessed and the range of injury severity [9, 13–16]. We used conventional coagulation plasma based assays to assess the degree of coagulopathy in our cohort. However, prothrombin time and aPTT assays only provide a rather incomplete assessment of a patient’s current haemostatic capacity [17]. Although viscoelastic testing, such as TEG and ROTEM, allows a more detailed analysis of the coagulation system in time, data based on this technology were only available in a small proportion of iTBI patients from the CENTER-TBI study core documentation, thus precluding meaningful analysis. For this reason, the conventional parameters INR, aPTT and platelet count were used as primary outcome marker indicating coagulopathy using the thresholds based upon previous studies [5, 18, 19].

The frequency of haemostatic alterations which occur in the context of iTBI may increase with injury severity [5, 9]. In the present study, AIS_Brain_ was not an independent predictor of coagulopathy; however, a larger proportion of patients with severe head injury (AIS_Brain_ ≥ 5) displayed alterations as compared to those with lower magnitudes sustained. Coagulopathy has previously been reported more frequently in penetrating than in blunt brain injuries [9, 19, 20]. In the present study, less than 2% of iTBI patients had sustained a penetrating injury mechanism. Therefore, the prevalence of coagulopathy reported corresponds rather to its prevalence in the context of a blunt injury mechanism.

Previous reports indicated that coagulopathic TBI patients had a nine times higher mortality and 30 times higher risk of unfavourable outcome compared to non-coagulopathic TBI patients [2, 9]. In the present cohort, a significant increase in mortality among coagulopathic iTBI patients (25.3%) compared to non-coagulopathic patients (9.0%) was observed. A retrospective study based upon a large dataset from trauma patients including those with TBI revealed that patients with blunt TBI showing at least one abnormality in their coagulation profile had a higher mortality rate than non-coagulopathic TBI patients [20]. In line with these findings, the coagulation parameters of iTBI patients in the present study without pre-injury anticoagulant and/or antiplatelet therapy were significantly deteriorated with increasing severity of brain injury, e.g. the higher the AIS_Brain_, the higher the INR and the lower the fibrinogen levels and platelet counts.

Anticoagulant and antiplatelet agents appear to worsen outcome in iTBI. For every fifth iTBI patient in the present study (*n *= 122), pre-injury intake of anticoagulant and/or antiplatelet agents was documented. Anticoagulant and/or antiplatelet drugs are increasingly prescribed for several indications in the elderly [21]. Vice versa, epidemiological studies have confirmed that the highest incidence of TBI occurs in older adults with falls as the most common mechanism leading to severe head injuries [22–24]. In particular, patients with pre-injury anticoagulant and/or antiplatelet drugs are at increased risk of developing a progressive haemorrhagic injury following a traumatic intracranial haemorrhage [5, 25–29]. In the present study, elderly iTBI patients with pre-injury anticoagulant and/or antiplatelet drugs had an almost twofold increased risk to establish haemostatic abnormalities than those without this risk factor (34% vs. 16%). It is conceivable that the increased haemostatic alteration risk in geriatric TBI patients is associated with pre-injury anticoagulant and/or antiplatelet therapy. In the present study, iTBI patients with pre-injury medication of vitamin K antagonists displayed a higher risk to develop an abnormal coagulation profile compared to those with other pre-injury anticoagulant and/or antiplatelet therapy. Most likely, these patients have an exacerbated progress of TBI, severe complications and outcome due to their pre-existing with vitamin K antagonists. In line with these findings, retrospective studies described higher prevalence of spontaneous bleeding rates and worse outcome in elderly, vitamin K-antagonist treated iTBI patients compared to other anticoagulant agents and platelet inhibitors [30–32]. Despite both groups having a median AIS_Brain_ = 4, haemostatic alteration was much more common among anticoagulated patients. If the risk factors for coagulopathy in iTBI patients with pre-injury anticoagulant and/or antiplatelet drugs were similar to those not on these drugs remain speculative due to the limited numbers of patients in these subgroups precluding a meaningful analysis. The overall outcomes among elderly iTBI patients on pre-injury anticoagulant and/or antiplatelet drugs in the present study were significantly worse compared to iTBI patients without anticoagulation therapy (mortality 35.2% in anticoagulated patients vs. 11.6% in non-anticoagulated patients).

Clinical data from prospective observational studies and meta-analyses on TBI patients have been used to describe factors that characterise the development of TBI-associated coagulopathy [2, 20, 33, 34]. The results of both uni- and multivariate analyses obtained from the present study identified hypotension, deranged BE, hypothermia, low GCS and hypoxia being associated with coagulopathy in iTBI patients. With an odds ratio of 3.51, hypotension was the most strongly associated risk factor identified. The results from an earlier prospective study showed that iTBI patients only developed a coagulopathy in the presence of a hypotension, regardless of head injury severity [35]. A base excess ≤ − 6 suggests tissue hypoperfusion most likely to result from systemic hypotension which had an odds ratio of 3.11 indicating coagulopathy. Hypothermia was further identified as an associated risk factor for acute coagulopathy following iTBI with OR of 2.89. In previous studies of trauma patients, hypothermia has been a risk factor for mortality but not directly for coagulopathy [36, 37]. Hypothermia induces coagulopathy by causing deterioration of platelet function, reducing activity of coagulation factors and reducing fibrinogen synthesis all together with increased morbidity and mortality [38–40]. Hypoxia plays an important role in worsening outcome in TBI as it may cause cerebral inflammation and the release of cytokines, augmenting further secondary brain injury [41, 42]. In the present study, hypoxia was identified as another risk factor indicating coagulopathy and poor outcome following iTBI (OR 2.09). In contrast to the univariate analysis (*p *= 0.016), hypoxia could not be statistically identified as risk factor in the multivariate analysis (*p *= 0.138). The difference in *p* values was marginal but exceeded *p *= 0.05. On the one hand, the variance of *p* values in the multivariate model was probably attenuated by correlation with other variables, hereby changing the effects (odds ratios) and *p* values of the other predictors. Thus, it may be that hypoxic patients showed other physical findings that may be captured in the model, so that the effect may differ from the univariate effect. On the other hand, hypoxia was observed in only 20 patients providing a further limitation leading to increased *p* values. Nevertheless, we consider that hypoxia is indeed a risk factor for coagulopathy, with an odds ratio of 2.09, but our data are not sufficient to prove this with 95% certainty.

Last but not least, GCS ≤ 8 at hospital admission was identified as an independent risk factor for acute coagulopathy in iTBI patients in this study. Other studies which have linked altered GCS with coagulopathy have proposed that injury to the brain itself may induce coagulation disturbances [5, 20, 43]. In a multivariate analysis of iTBI patients from the German Trauma Registry (TR-DGU^®^), a low GCS (≤ 8) was identified as an independent risk factor for coagulopathy after TBI [20]. It was also concluded that a lower GCS may correlate with a higher risk of neurological decline in iTBI patients with coagulopathy [20]. Related to the identified risk factors, we cannot exclude volume substitution as well as receipt of blood products or haemostatic agents during early prehospital care as a potential cofounder that may have altered haemostatic capacity in the severely injured patients, as a prehospital data collection was not part of the CENTER-TBI core study. Likewise, early in-hospital blood product administration prior to any laboratory coagulation testing was marginally evaluated and precluded a more detailed analysis at this stage. The predictors identified in this study could be used in clinical settings to identify high-risk patients earlier. The results could also support in defining the course and the severity of coagulopathy following iTBI.

### Limitations

The present study is the first report on haemostatic alterations occurring in the context of iTBI based upon data from the longitudinal, observational CENTER-TBI core study cohort. The results confirm previous findings on demographics, clinical presentation and coagulation status during the acute phase, e.g. within 4 h, after iTBI. Future analyses will now more thoroughly investigate the coagulation abnormalities encountered in this unique and highly detailed patient dataset. The limitations to the given study apart from those inherent to retrospective analysis of a large prospectively collected dataset include that the recruitment to the CENTER-TBI core study was not consecutive and was determined by site logistics and research interests. This means that patient selection bias may be possible. Likewise, coagulation parameters beyond those used for conventional testing, in particular those potentially reflecting functional deficits, were only marginally captured and precluded more in-depth analysis at this stage. This also refers to the completeness of the datasets analysed as data collection was performed over 4 years. However, among variables considered for this analysis, there was little missing data. The reported associations remain purely descriptive. It can certainly not be concluded from the present analysis whether the observed coagulopathy was the result of the iTBI itself or the precipitating factor that led to a worsening of the clinical situation along with iTBI.

## Conclusion

The prevalence of coagulopathy in iTBI patients on pre-injury anticoagulant and/or antiplatelet therapy was significantly higher than in patients without anticoagulant therapy. Independent risk factors associated with acute coagulopathy in iTBI included systolic hypotension, base excess, hypothermia, reduced GCS on ED admission and hypoxia. The acknowledgement and assessment of these risk factors could be helpful in clinical practice for the early identification of TBI-associated coagulopathy, resulting in the expeditious provision of appropriate, targeted clinical management. It remains to be determined whether to coagulopathy seen was the result of the iTBI itself or a precipitating factor for neuroworsening.
